# Mean Platelet Volume-to-Platelet Count Ratio (MPR) in Acute Exacerbations of Idiopathic Pulmonary Fibrosis: A Novel Biomarker for ICU Mortality

**DOI:** 10.3390/medicina61020244

**Published:** 2025-01-31

**Authors:** Maside Ari, Berna Akinci Ozyurek, Murat Yildiz, Tarkan Ozdemir, Derya Hosgun, Tugce Sahin Ozdemirel, Kerem Ensarioglu, Mahmut Hamdi Erdogdu, Guler Eraslan Doganay, Melek Doganci, Oral Mentes, Omer Faruk Tuten, Deniz Celik

**Affiliations:** 1 Department of Pulmonology, Ankara Ataturk Sanatorium Training and Research Hospital, 06290 Ankara, Turkey; drberna_1982@yahoo.com (B.A.O.); drmuratyildiz85@gmail.com (M.Y.); deryahosgun@gmail.com (D.H.); drtugcesahin@gmail.com (T.S.O.); kerem.ensarioglu@gmail.com (K.E.); mahmuthamdierdogdu@gmail.com (M.H.E.); 2 Department of Pulmonology, Konya Farabi Hospital, 42090 Konya, Turkey; tarkanozdemir78@gmail.com; 3 Department of Anesthesiology and Reanimation, Ankara Ataturk Sanatorium Training and Research Hospital, 06290 Ankara, Turkey; gulerdoganay@hotmail.com.tr (G.E.D.); melekdidik@hotmail.com (M.D.); 4 Intensive Care Unit, Ankara Gulhane Training and Research Hospital, 06010 Ankara, Turkey; omentes@live.com; 5 Clinic of Lung Diseases, Health Practice and Research Hospitals, Department of Pulmonology, Faculty of Medicine, Ankara University, 06230 Ankara, Turkey; omertuten@gmail.com; 6 Department of Pulmonology, Faculty of Medicine, Alanya Alaaddin Key Kubat University, Education and Research Hospital, 07450 Antalya, Turkey; drdenizcelik@hotmail.com

**Keywords:** IPF-AE, mortality, MPR, platelet, ICU

## Abstract

*Background and Objectives*: Acute exacerbation of idiopathic pulmonary fibrosis (IPF-AE) often results in severe respiratory distress requiring treatment in the intensive care unit and has a high mortality rate. Identifying prognostic markers and assessing disease severity are crucial for clinicians to gain detailed insights. The mean platelet volume-to-platelet count ratio (MPR) is an inflammatory marker commonly used in malignancies. This study aimed to evaluate MPR and other factors affecting mortality in patients with IPF-AE who were monitored in the intensive care unit (ICU). *Materials and Methods*: This retrospective study was conducted on patients monitored in the ICU for IPF-AE between 2017 and 2023. Demographic characteristics, vital signs, laboratory and imaging findings, and administered treatments were reviewed. MPR was calculated by dividing the mean platelet volume by the platelet count. The primary endpoint was defined as 1-month in-hospital mortality. *Results*: A total of 59 patients monitored in the ICU for IPF-AE were included in the study. The mean age of the patients was 62.75 years, and 81.4% of the participants were male. During the 30-day follow-up period, 62.7% of the patients died. The need for invasive mechanical ventilation (IMV) was significantly associated with increased mortality (*p* < 0.001). The optimal cutoff value for MPR was determined to be 0.033, with a sensitivity of 83.7% and specificity of 63.64%, indicating its predictive value for mortality (AUC: 0.764; 95% CI: 0.635–0.864; *p* < 0.001). *Conclusions*: In this study, the need for IMV emerged as a critical parameter in predicting mortality in patients with IPF-AE. Additionally, the use of the MPR as a prognostic biomarker may offer a novel approach in the management of IPF patients. These findings could contribute to the development of strategies aimed at early intervention in IPF patients. Further studies with larger sample sizes are needed to validate these results. This study has demonstrated that MPR is a significant prognostic biomarker for predicting mortality in patients with IPF-AE who are managed in the intensive care unit. The potential use of MPR as a biomarker in clinical decision-making may provide new approaches to the management of IPF patients. Additionally, the need for IMV in IPF-AE emerges as a critical parameter for predicting mortality. These findings may contribute to the development of early intervention strategies for IPF patients. Further studies with larger cohorts are needed to validate these results.

## 1. Introduction

Idiopathic pulmonary fibrosis (IPF) is a chronic, progressive, fibrotic interstitial lung disease. In untreated patients who are not candidates for lung transplantation, the average life expectancy is 2–3 years [1]. The potential of antifibrotic therapies to slow the progression of IPF has been demonstrated in the literature [2]. Large-scale cohort studies evaluating both drugs have shown that IPF treatment generally improves efficacy regardless of age, gender, or ethnicity [3]. The clinical course of IPF is unpredictable and varies among patients [4]. While most patients are managed in outpatient care settings, approximately 10% experience acute exacerbations (AEs) within the first two years following diagnosis [5]. Acute exacerbation of idiopathic pulmonary fibrosis (IPF-AE) is typically characterized as clinically significant respiratory deterioration developing within less than a month, associated with new diffuse alveolar damage. The annual incidence of IPF-AE is estimated to be 4.1%. Acute exacerbations are the leading cause of mortality in IPF patients. Despite advances in medical therapies, in-hospital mortality rates exceed 50% [6]. Furthermore, post-discharge mortality within the first six months remains notably high. Identifying prognostic factors in these patients is critical for effective patient management.

Complete blood count (CBC) is a routine laboratory test with prognostic significance in many diseases. A recent study on IPF patients evaluated CBC parameters, highlighting the prognostic value of red cell distribution width and the neutrophil-to-lymphocyte ratio [7]. CBC includes mean platelet volume (MPV) and platelet count (PC). Platelets have been shown to play a significant role in the immune response to inflammation [8]. MPV reflects platelet size and activity and has been evaluated in diseases with intense inflammation, being reported as an important biomarker for predicting survival [9,10]. A strong negative correlation exists between MPV and PC. The mean platelet volume-to-platelet count ratio (MPR) was first proposed as a survival marker in pancreatic cancer [11] and has since been identified as a predictive biomarker for prognosis in various diseases [12,13]. Considering the role of platelets in IPF, the heightened inflammation observed during acute exacerbations could make MPR even more significant. However, the relationship between MPR and IPF-AE has not yet been reported in the literature. Therefore, this study aimed to evaluate MPR and other factors affecting mortality in patients with IPF-AE monitored in the intensive care unit.

## 2. Materials and Methods

This study was retrospectively designed to include patients diagnosed with IPF-AE who were monitored in the intensive care unit (ICU) of our hospital between 1 January 2017 and 15 November 2023. IPF diagnosis date, treatments used for IPF with computed tomography, and demographic characteristics were examined. Vital findings at the time of admission to intensive care, blood tests taken within the first 24 h, treatments applied, respiratory support, and outcome status were recorded.

The diagnosis of IPF-AE was based on the following criteria:

1. A prior or current diagnosis of IPF.
2. Acute worsening of dyspnea within less than one month.
3. Presence of new bilateral ground-glass opacities and/or consolidations superimposed on an underlying usual interstitial pneumonia (UIP) pattern on thoracic CT.
4. Worsening not fully explained by heart failure or fluid overload.

Patients meeting these criteria were classified as having IPF-AE after excluding alternative diagnoses such as pneumonia, pulmonary embolism, and pneumothorax.

The extent of radiological involvement in patients was assessed using high-resolution computed tomography (HRCT). HRCT was performed in the supine position during inspiration and expiration, with 1.0 mm thick sections covering the entire lungs. The imaging evaluations included assessment of upper lobe involvement and the extent of disease involvement. The CT images of the patients included in our study were independently evaluated by two pulmonologists and one radiologist.

In our research, CBC analyses were performed using the Mindray BC 6800 automated complete blood count analyzer (Shenzhen Mindray Bio-medical Electronics Co., Ltd., Shenzhen, China). PCs were determined using the impedance method, which involves directly counting the platelets passing through the aperture, with results expressed in units of 10^3^/µL. MPV was simultaneously derived from the platelet histogram.

MPV and PC were obtained from the complete blood count performed within the first 24 h of ICU admission. The MPR was calculated by dividing the MPV by the PC.

### 2.1. Patient Selection

Patients monitored in the ICU for IPF-AE during the specified dates were included in the study. Exclusion criteria were as follows: patients with any history of platelet or hematopoietic abnormalities related to MPV or platelet, patients under 18 years of age, those monitored for less than 24 h, patients with IPF who were admitted for reasons other than acute exacerbation, patients electively admitted to the ICU for postoperative monitoring, patients with interstitial lung diseases other than IPF, patients diagnosed with disseminated intravascular coagulation (DIC), and those with incomplete data.

Inclusion Criteria

- Diagnosis of IPF.
- Patients managed in the intensive care unit with IPF-AE.
- Patients with a history of any platelet or hematopoietic abnormality related to MPV and platelets.
- Patients under 18 years of age.

Exclusion Criteria

- Patients with IPF who were managed for reasons other than acute exacerbation.
- Patients with interstitial lung diseases other than IPF.
- Patients admitted to the intensive care unit electively following a surgical procedure.
- Patients with incomplete clinical data.
- Patients with incomplete radiological data.
- Patients with a hospital stay of less than 24 h.
- Patients diagnosed with disseminated intravascular coagulation (DIC).

**Figure 1:**
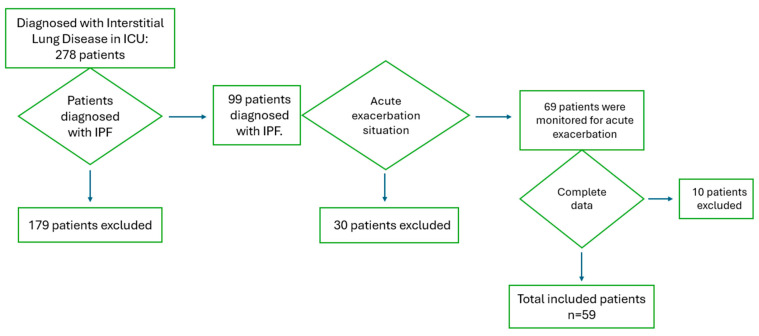
Flowchart of patients included in and excluded from this study.

Figure 1 shows a flowchart detailing the patients included in and excluded from this study.

Our hospital serves as a tertiary care reference center for pulmonary diseases. Following the diagnosis of an IPF-AE, patients are managed and treated in the pulmonology ward if ICU admission is not deemed necessary after clinical evaluation. Patients requiring intensive care are managed in our ICU facilities.

In the presence of appropriate findings for the diagnosis of DIC, the DIC scoring system developed by the International Society on Thrombosis and Haemostasis was used. The total score was calculated by combining platelet count, fibrinogen levels, prolongation of prothrombin time (PT), and D-dimer levels. A score greater than 5 was considered indicative of a high likelihood of DIC, and consultation with the hematology department was obtained. Patients suspected of having DIC were excluded from the study. Ethical approval for the study was obtained from the Clinical Research Ethics Committee of our hospital, with decision number 2840, dated 22 November 2023. Ethical principles outlined in the Declaration of Helsinki were adhered to throughout the study.

### 2.2. Statistical Analysis

The statistical analysis of the collected data was performed using IBM SPSS Statistics version 27.0. The normality of the distribution of continuous variables was evaluated using the Kolmogorov–Smirnov test. The data following a normal distribution were presented as mean ± standard deviation (Mean ± SD), while the data not following a normal distribution were presented as median (min-max). The categorical variables were expressed as frequencies and percentages (%), and continuous variables were reported as Mean ± SD or median (min-max), depending on their distribution. The categorical variables between groups were compared using the Chi-square test, and continuous variables were compared using the Independent T Test (for normally distributed data) or the Mann–Whitney U test (for non-normally distributed data), depending on the distribution of the data.

A receiver operating characteristic (ROC) analysis was performed for the MPR laboratory parameter. The analysis determined the optimal cut-off point for predicting mortality, and the associated Area Under the Curve (AUC), sensitivity, specificity, positive predictive value (PPV), negative predictive value (NPV), as well as positive and negative likelihood ratios (LR+ and LR−) were calculated.

Univariate Cox Regression Analysis was used to evaluate the relationship between clinical, radiological, and laboratory parameters and mortality. The results of the univariate analysis were reported as Hazard Ratios (HR), 95% Confidence Intervals (CI), and *p*-values. The parameters found to be significant in the univariate analysis were included in the multivariate Cox regression analysis to control for the effects of other factors. In the multivariate analysis, the independent effects of these parameters on mortality were evaluated as independent risk factors, and the results were presented with HR, 95% CI, and *p*-values. Comparisons with a *p*-value below 0.05 were considered statistically significant.

## 3. Results

Among the patients monitored in the intensive care unit, 278 were diagnosed with interstitial lung disease. Of these, 99 patients had a diagnosis of IPF. Thirty patients who were monitored for reasons unrelated to an acute exacerbation and ten patients with incomplete data were excluded from the study. A total of 59 patients were included in the analysis. The demographic characteristics of the included patients are presented in Table 1.

A total of 45 patients included in the study were using antifibrotic therapy. The clinical characteristics of IPF and treatments administered to the patients are presented in Table 2.

When examining clinical factors associated with mortality in the included patients, the need for invasive mechanical ventilation was found to be significantly associated with increased mortality (*p* < 0.001). Mortality was also higher in patients with upper lobe involvement observed on computed tomography (*p* = 0.043) (Table 3).

Table 4 presents the evaluation of laboratory findings at the time of admission, providing a comprehensive overview of key parameters assessed during the initial examination.

**Figure 2:**
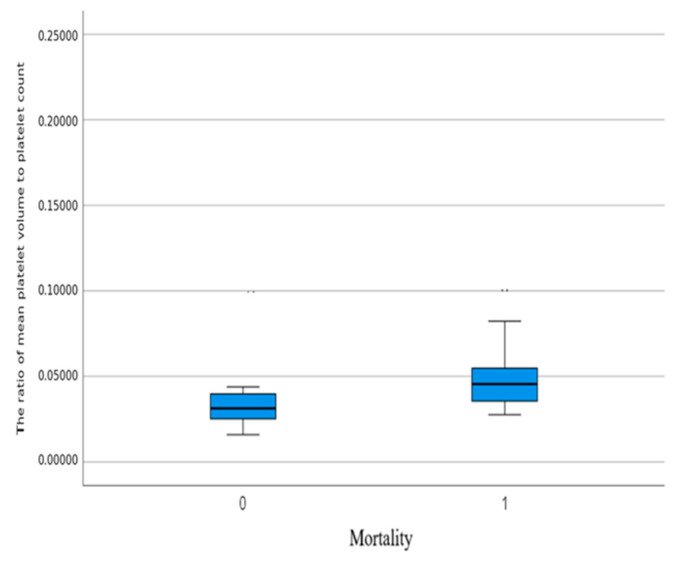
Distribution of the Mean Platelet Volume-to-Platelet Count Ratio in Surviving and Deceased Patients.

MPR was found to be 0.043 in surviving patients and 0.048 in deceased patients. A significant difference was observed between the groups (*p* < 0.001) (Figure 2).

**Figure 3:**
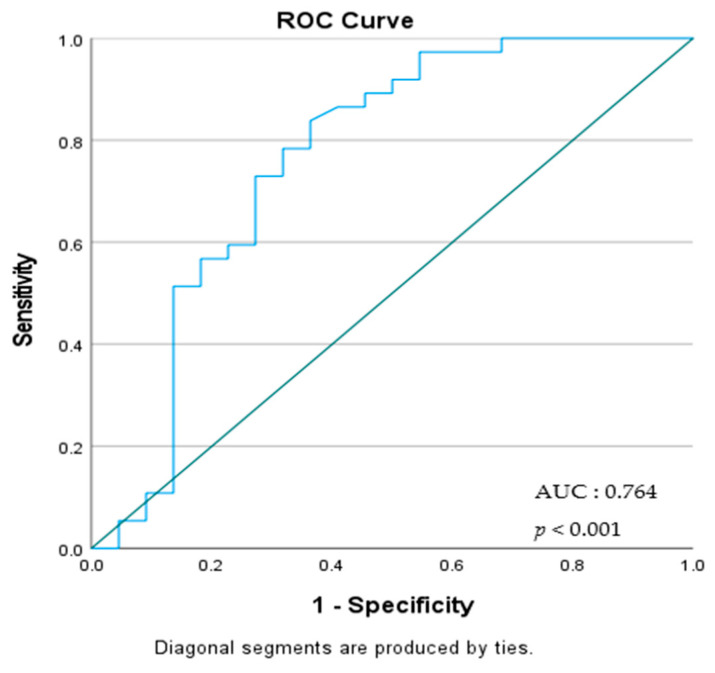
ROC Analysis of the Mean Platelet Volume-to-Platelet Count Ratio in Predicting Mortality.

The cut-off value for MPR was determined to be 0.033, with a sensitivity of 83.7% and a specificity of 63.64%, and it was identified as a significant predictor of mortality (AUC: 0.764, 95% CI: 0.635–0.864; *p* < 0.001) (Table 5). The ROC curve for MPR in predicting mortality is shown in Figure 3.

To assess the independent effects of clinical, radiological, and laboratory parameters on mortality, the impact of dependent variables and MPR was initially examined. During this process, the two most significant parameters were identified. Using these parameters, a multivariate Cox regression model was developed to provide a more comprehensive evaluation of the independent effect of MPR on mortality alongside other variables. The results are presented in Table 6.

In univariate analysis, upper lobe involvement on computed tomography was significantly associated with mortality (HR: 2.13, 95%CI: 1.06–4.27, *p* = 0.032). Additionally, platelet count (HR: 0.996, 95%CI:0.002–0.999, *p* = 0.010) and MPV (HR: 1.44, 95%CI: 1.05–1.96, *p* = 0.022) were also significantly associated with mortality.

A MPR > 0.033 was identified as a significant risk factor in univariate analysis (HR: 4.55, 95%CI: 1.76–11.78, *p* = 0.002). However, it did not reach statistical significance in the multivariate analysis (HR: 2.17, 95%CI: 0.79–5.92, *p* = 0.130).

Invasive mechanical ventilation support was identified as the strongest independent risk factor for mortality in both the univariate (HR: 13.23, 95%CI: 4.01–43.64, *p* < 0.001) and multivariate analyses (HR: 9.98, 95% CI: 2.92–34.12, *p* < 0.001).

## 4. Discussion

This study presents an evaluation of 59 patients diagnosed with IPF who were monitored in the ICU. The factors affecting mortality were investigated in light of the patients’ clinical, radiological, and laboratory findings. The results obtained through Cox regression analysis demonstrated that certain clinical parameters, particularly the need for invasive mechanical ventilation (IMV), significantly increased the mortality risk of the patients. Additionally, radiological findings, such as upper lobe involvement on computed tomography, as well as laboratory parameters including PC, MPV, and the MPR, were identified as valuable prognostic indicators for predicting mortality. Our study highlights the potential clinical utility of MPR, which could be validated further through larger cohort studies and prospective research.

This study presents an evaluation of 59 patients diagnosed with IPF who were managed in the intensive care unit. It is one of the first studies to assess the prognostic significance of the MPR in patients with IPF-AE. Our findings indicate that MPR can serve as a significant predictor of mortality. Consistent with previous studies linking MPR to mortality, our results support the prognostic potential of this parameter in IPF patients. Given the retrospective design of our study, the integration of MPR into clinical practice should be further validated through larger and prospective studies. Additionally, the need for IMV significantly increases the mortality risk in patients.

Mortality is high in IPF-AE cases requiring ICU admission due to acute respiratory failure (ARF). However, reported mortality rates vary across studies. In general, mortality is influenced by the subtype of interstitial lung disease (ILD), with IPF being associated with worse outcomes compared to other ILD subtypes [14]. In a study by Ba et al. on patients with ILD monitored for respiratory failure, mortality was reported to be higher in IPF compared to other subtypes, with in-hospital mortality exceeding 50% [15]. In our study, in-hospital mortality was found to be 67.4%.

In our study, 81.4% of the included patients were male. The most common comorbidities were cardiovascular diseases. It was observed that the majority of patients (76.2%) were on antifibrotic therapy. A recent review examining the clinical characteristics and treatment strategies of IPF-AE highlighted that the pathogenesis of IPF-AE remains unclear and that multiple mechanisms may be involved simultaneously. Corticosteroids were reported to remain the mainstay of treatment for IPF-AE [16]. Similarly, in our study, all patients received corticosteroid therapy.

In IPF, the development of ARF requiring IMV is associated with poor prognosis. The literature reports that mortality is very high in acute exacerbations associated with ILD requiring intensive care [17]. A recent study also demonstrated that in fibrosis-dominant ILD patients, mortality reaches up to 75% in cases requiring mechanical ventilation [18]. In our study, the majority of patients (67.8%) required invasive mechanical ventilation. The analysis showed a strong association between the need for IMV and mortality (*p* < 0.001).

The course of IPF is unpredictable; however, given its potentially aggressive progression, radiological extent is of great importance in predicting mortality. In IPF, the honeycombing pattern typically has a basal and peripheral predominance. Upper lobe involvement may be observed in the advanced stages of the disease. Another significant finding in our study was that patients with upper lobe involvement on computed tomography had higher mortality rates (*p* = 0.043). This finding is meaningful as it may be associated with more advanced disease. The impact of upper lobe involvement on mortality was demonstrated in univariate regression analysis. However, in multivariate regression, its significance appeared to diminish, likely due to the dominant effect of other factors, such as the need for invasive mechanical ventilation support.

In IPF, increased platelet activation and accumulation in the lung fields have been demonstrated in animal studies [19]. MPV has been reported to be elevated in IPF patients compared to healthy controls and may serve as a marker of platelet activation [20]. However, no direct causal relationship between platelets or platelet-derived products and IPF progression has been established in studies. In our study, thrombocytopenia was found to be associated with mortality in these critically ill IPF patients. Additionally, higher MPV levels were observed in patients with fatal outcomes.

MPR was first proposed as a survival marker in pancreatic cancer [11]. It has since been evaluated as a predictive marker for prognosis in various diseases [21,22]. In IPF-AE, the increased severity of inflammation further accentuates the prognostic value of MPR. In our study, the use of MPR for mortality prediction was validated through ROC analysis. With the determined cut-off value for MPR (83.7% sensitivity, 63.64% specificity), it emerged as a significant indicator for predicting mortality. MPR was identified as a significant risk factor in univariate analysis; however, it did not reach statistical significance in multivariate analysis. This finding was associated with the need for IMV, which is another parameter in multiple regression, being a very strong independent risk factor for mortality. Our study can serve as a guide for further exploration of MPR’s effectiveness in clinical practice, requiring validation and more detailed investigation through larger cohort studies and prospective research. This finding underscores the importance of MPR as a biomarker reflecting the severity of inflammation and platelet activation. However, the loss of significance of MPR in multivariate analysis may be attributed to the influence of stronger prognostic factors, such as invasive mechanical ventilation. The utility of MPR as a prognostic tool in clinical practice should be further investigated through large-scale cohort studies and prospective research. In this context, our study serves as a pioneering work, highlighting the potential significance of MPR in predicting mortality in IPF-AE.

Our study has certain limitations. IPF is a rare disease, and with the introduction of antifibrotic therapies, acute exacerbations have become less common. This is thought to have contributed to the small sample size of our study. Additionally, respiratory function test (RFT) findings were not available for all patients, and compliance with RFTs was limited in end-stage patients. Therefore, RFT findings were not included in the analysis.

Our study has some limitations. As the study was conducted retrospectively, the collection of data in a retrospective manner may have limited access to certain clinical information. Additionally, the study was carried out at a single center, which may restrict the generalizability of the results. Validation of these findings through studies conducted at multiple centers is required. IPF is a rare disease, and the widespread use of antifibrotic therapies has reduced the frequency of acute exacerbations, leading to a limited number of patients included in the study. Furthermore, pulmonary function test (PFT) results were not available for all patients, and PFT compliance was limited in patients at advanced stages of the disease. Therefore, PFT findings were not included in this study.

## 5. Conclusions

In our study of patients monitored in the intensive care unit for IPF-AE, we found that age and gender did not influence prognosis of acute exacerbations. However, the need for invasive mechanical ventilation and the radiological extent of the disease were associated with increased mortality. Additionally, the use of the MPR as a prognostic biomarker may offer a novel approach in the management of IPF patients. These findings could contribute to the development of strategies for early intervention in IPF patients. Further studies with larger sample sizes are needed to validate these results.

This study has demonstrated that MPR is a significant prognostic biomarker for predicting mortality in patients with IPF-AE managed in the intensive care unit. 

## Figures and Tables

**Table 1:** Demographic Characteristics of the Included Patients.

All Patients (N = 59)	
Age, years (Mean ± SD)	62.75 ± 11.11
Gender	
Female	11 (18.6%)
Male	48 (81.4%)
Comorbidities (N (%))	46 (78%)
Cardiovascular disease	23 (39%)
Diabetes mellitus	20 (33.9%)
Neurological disease	2 (3.4%)
Chronic obstructive pulmonary disease	10 (16.9%)
Malignancy	7 (11.9%)

**Table 2:** Clinical Characteristics of the Included Patients.

All Patients (N = 59)	
Use of Antifibrotic Therapy	45 (76.2%)
Pirfenidone	27 (45.7%)
Nintedanib	18 (30.5%)
Use of Immunosuppressive Therapy	3 (0.5 %)
Time from IPF * Diagnosis to ICU ** Admission (months, Mean ± SD)	36 ± 30
Upper Lobe Involvement on Computed Tomography	14 (23.7%)
Antibiotic Therapy Usage	59 (100%)
Steroid Usage	59 (100%)
Respiratory Support	
Invasive Mechanical Ventilation	38 (64.4%)
SOFA Score	8.57 ± 2.60
APACHE-II Score	21.18 ± 8.33

* Idiopathic Pulmonary Fibrosis. ** Intensive Care Unit.

**Table 3:** Characteristics of Patients by Mortality Status.

Characteristic	Surviving Patients (N = 22, 37.3%)	Deceased Patients (N = 37, 62.7%)	*p*-Value
Age, years (Mean ± SD)	62.68 ± 13.66	62.78 ± 9.46	0.680
Gender			
Female	5 (22.7%)	6 (16.2%)	0.538
Male	17 (77.3%)	31 (83.8%)	
Presence of Comorbidities	19 (86.4%)	27 (73%)	0.234
Upper Lobe Involvement on CT	2 (9.1%)	12 (32.4%)	0.043
Need for Invasive Mechanical Ventilation	4 (18.2%)	34 (91.9%)	<0.001
SOFA Score	6.18 ± 1.94	10.00 ± 1.76	<0.001
APACHE-II Score	13.27 ± 4.38	25.89 ± 6.30	<0.001

**Table 4:** Evaluation of Laboratory Findings at Admission.

Laboratory Findings	All Patients (N = 59) (Min–Max)	Surviving Patients (N = 22) (Min–Max)	Deceased Patients (N = 37) (Min–Max)	*p*-Value
CRP (mg/L) *	106 (4–322)	103 (7–293)	87 (7–196)	0.175
Platelet Count (×10^3^/µL)	252 (37–595)	294 (37–595)	226 (96–348)	0.004
Mean Platelet Volume (%)	9.69 (7.7–12.4)	9.29 (7.7–11.8)	9.93 (8.1–12.4)	0.007
MPR **	0.046 (0.015–0.220)	0.043 (0.015–0.220)	0.048 (0.027–0.105)	<0.001

* CRP: C-Reactive Protein. ** MPR: Mean Platelet Volume-to-Platelet Count Ratio.

**Table 5:** ROC Analysis Results for the Predictive Value of the MPR * in Mortality.

Parameter	AUC	95% Confidence Interval	Cut-off Value	Sensitivity (%)	Specificity (%)	PPV (%)	NPV (%)	LR+	LR−	*p*-Value
MPR *	0.764	0.635–0.864	0.033	83.78	63.64	79.5	70.0	2.30	0.25	<0.001

* MPR: Mean Platelet Volume-to-Platelet Count Ratio.

**Table 6:** Cox Regression Analysis Results for Factors Associated with Mortality.

Variable	Univariate Cox Regression		Multivariate Cox Regression	
	HR (95% CI)	*p*-Value	HR (95% CI)	*p*-Value
Upper lobe involvement on CT	2.13 (1.06–4.27)	0.032	1.20(0.58–2.47)	0.610
Platelet count	0.996 (0.002–0.999)	0.010		
MPV *	1.44 (1.05–1.96)	0.022		
MPR ** > 0.033	4.55 (1.76–11.78)	0.002	2.17(0.79–5.92)	0.130
Invasive mechanical ventilation support	13.23 (4.01–43.64)	<0.001	9.98(2.92–34.12)	<0.001

* MPV: Mean Platelet Volume. ** MPR: Mean Platelet Volume-to-Platelet Count Ratio.

## Data Availability

The original contributions presented in this study are included in the article. Further inquiries can be directed to the corresponding author.
